# Executioner caspases and CAD are essential for mutagenesis induced by TRAIL or vincristine

**DOI:** 10.1038/cddis.2017.454

**Published:** 2017-10-05

**Authors:** Mark A Miles, Christine J Hawkins

**Affiliations:** 1 Department of Biochemistry and Genetics, La Trobe Institute for Molecular Science, La Trobe University, Bundoora, VIC, Australia

## Abstract

Chemotherapy drugs interfere with cellular processes to generate genotoxic lesions that activate cell death pathways. Sustained DNA damage induced by these drugs can provoke mutations in surviving non-cancerous cells, potentially increasing the risk of therapy-related cancers. Ligation of death receptors by ligands such as TRAIL, and subsequent activation of extrinsic apoptotic pathways, also provokes mutations. In this study, we show that executioner caspase activation of the apoptotic nuclease CAD/DFF40 is essential for TRAIL-induced mutations in surviving cells. As exposure to chemotherapy drugs also activates apoptotic caspases and presumably CAD, we hypothesized that these pathways may also contribute to the mutagenesis induced by conventional chemotherapy drugs, perhaps augmenting the mutations that arise from direct DNA damage provoked by these agents. Interestingly, vincristine-mediated mutations were caspase and CAD dependent. Executioner caspases accounted for some of the mutations caused by the topoisomerase poisons doxorubicin and SN38, but were dispensable for mutagenesis following treatment with cisplatin or temozolomide. These data highlight a non-apoptotic role of caspases in mutagenesis mediated by death receptor agonists, microtubule poisons and topoisomerase inhibitors, and provide further evidence for a potential carcinogenic consequence of sublethal apoptotic signaling stimulated by anticancer therapies.

Conventional chemotherapy agents provoke DNA damage and/or perturb mitosis in order to trigger apoptotic pathways in tumor cells, to eliminate the patient’s cancer.^1^ Platinating agents interact with DNA to form monoadducts and strand crosslinks,^2^ whereas alkylating agents introduce alkyl groups to nucleotides to cause DNA-adducts and interstrand crosslinks.^3^ During replication, topoisomerases alleviate helical stress by inflicting transient single-strand (topoisomerase-I) or double-strand (topoisomerase II) breaks in DNA; topoisomerase poisons stabilize DNA-topoisomerase complexes to maintain these strand breaks.^4^ Some topoisomerase inhibitors also act as DNA intercalating agents.^5^ Microtubule-targeting agents block mitosis by suppressing spindle-microtubule dynamics, resulting in mitotic catastrophe leading to apoptosis.^6^ The disruption of DNA replication or progression through the cell cycle by these drugs results in the accumulation of double-strand DNA breaks. If DNA damage is too extensive for repair, cells undergo intrinsically activated apoptosis, modulated by p53 and the Bcl-2 family of proteins, to promote mitochondrial outer membrane permeabilization (MOMP) and caspase activation via the apoptosome.^7^ Unfortunately, a number of these genotoxic lesions promote the activation of error-prone DNA repair mechanisms and surviving cells may acquire mutations.^8^ Therefore, mutagenic anticancer drugs may possess oncogenic potential and may promote the development of subsequent 'therapy-related' cancers in cured patients.^9^

Some classes of drugs that can directly engage components of the apoptotic pathway, such as those targeting IAP proteins, fail to provoke mutations in surviving cells^10^ implying that these drugs may reduce the risk of second malignancies in cancer survivors. However, death receptor agonists, such as tumor necrosis factor-related apoptosis-inducing ligand (TRAIL/Apo2L), which directly activate extrinsic apoptosis, were mutagenic.^11^ Ligation of TRAIL death receptors upon binding of the TRAIL ligand promotes the recruitment of FADD and caspases-8 and/or -10 to the cytoplasmic death inducing signaling complex (DISC), activating these initiator caspases.^12^ Subsequent activation of executioner caspases-3 and -7 results either from direct cleavage by caspase-8 ('type I' cells) or via cleavage of the BH3-only protein Bid to stimulate Bax-/Bak-dependent MOMP ('type II' cells).^13^ TRAIL and agonistic antibodies targeting TRAIL receptors have progressed into early phase clinical trials but have failed to show robust antitumor activity in most patients.^14^ Hopefully further developments in TRAIL-based delivery and patient selection will potentiate the clinical use of these agents.

Executioner caspases can cleave an array of target proteins leading to characteristic apoptotic breakdown of a cell.^15^ DNA fragmentation is a caspase-mediated hallmark of apoptosis, which helps facilitate the clearance of apoptotic bodies.^16^ Caspase-activated DNase (CAD/DFF40) is the primary nuclease responsible for apoptotic DNA fragmentation.^17, 18^ Following its translation, CAD exists in an inactive heterodimeric complex with its inhibitor and chaperone, ICAD/DFF45, but caspases can cleave ICAD to release active CAD.^17, 18, 19^ Although caspases-3 and -7 cleave ICAD most potently, caspase-8 could also process this substrate with reduced efficiency.^20, 21^ CAD preferentially cleaves double-stranded DNA to generate blunt ends or ends with single-base overhangs.^22, 23^

Apoptotic DNA fragmentation has been implicated in promoting therapy-related leukemias in contexts where sublethal apoptotic signaling activates CAD in cells that maintain viability. Cleavage of a region of the mixed myeloid lineage (MLL) gene could be induced by drugs that target topoisomerases or (to a lesser extent) by apoptotic stimuli including death ligands.^24, 25, 26, 27^ The DNA-strand breaks within MLL appeared to involve error-prone repair via non-homologous end joining (NHEJ), which may contribute to the high incidence of chromosomal translocations associated with therapy-related acute myeloid leukemia.^28^ CAD has been directly linked to DNA damage in various contexts. Caspases were essential for CAD-mediated DNA damage in cells undergoing prolonged mitotic arrest^29, 30^ or in cells experiencing a low level of MOMP.^31^ Caspase-3 induced DNA damage via CAD in differentiating myoblast cells^32^ and was implicated in the induction of senescence.^30, 33^

Using siRNA-mediated transient CAD downregulation, we previously implicated CAD in TRAIL-induced DNA damage.^11^ Proteins involved in NHEJ seemed essential for repair of TRAIL-induced DNA damage^34^ and repair of fragmented DNA often involves NHEJ.^35^ Deletions were the most frequent class of TRAIL-induced mutations, consistent with NHEJ-mediated mis-repair.^36^ However, caspase-mediated activation of a different nuclease, Endonuclease G (EndoG) was responsible for DNA damage observed after *γ*-radiation exposure.^37^

This study was designed to conclusively ascertain the role of CAD in causing not only TRAIL-induced DNA damage, but also mutations in surviving cells, and to define the essential caspases within the TRAIL mutagenesis pathway. Phosphorylation of the H2AX protein (*γ*H2AX) provides an indication of DNA damage,^38^ however, a significant proportion of cells experiencing DNA damage may be destined to die. We therefore used the hypoxanthine–guanine phosphoribosyltransferase (HPRT) gene mutation assay to measure drug-induced mutagenesis in clonogenically competent cells. Cells with functional HPRT activity die in the presence of the toxic purine analog 6-thioguanine (6TG), whereas cells bearing HPRT loss-of-function mutations are 6TG resistant, so survive and proliferate.^39^ This study also examined the contributions of caspases and/or CAD to the mutagenesis provoked by conventional chemotherapy drugs. Our data confirmed that CAD and caspases are essential to the mutagenic function of TRAIL, and further revealed that this pathway is also responsible for vincristine-mediated mutagenesis. Caspases accounted for a small proportion of the mutations stimulated by topoisomerase poisons, but neither caspases nor CAD were required for mutagenesis induced by other classes of chemotherapy drugs.

## Results

### CAD is responsible for TRAIL mutations

**Figure 1:**
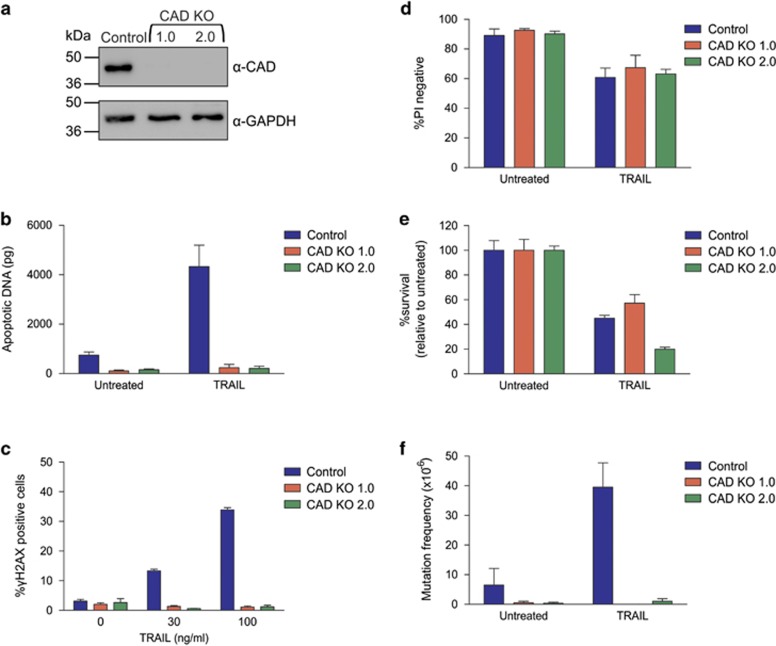
TRAIL is only mutagenic when CAD is expressed. (**a**) CAD protein expression in parental CAD expressing (control) and CAD knockout cells was assessed by immunoblot. The blot was reprobed with an antibody to GAPDH to indicate loading. (**b**) Cells were treated with no drug or 300 ng/ml TRAIL for 24 h then harvested. DNA was extracted for DNA fragmentation analysis by LM-qPCR detection. (**c**) Cells were treated with TRAIL for 5 h and the proportion of *γ*H2AX-positive cells was quantitated by flow cytometry. Cells were treated with 300 ng/ml TRAIL for 24 h then harvested. Propidium iodide was added to some cells to determine acute cell death (**d**), whereas clonogenicity assays were performed on other cells to determine the proportion of cells maintaining clonogenic competency after treatment (**e**). Surviving cells were grown in 6TG to select for the emergence of any HPRT mutants (**f**). Error bars represent mean±S.E.M. from three independent experiments

To determine whether CAD is required for TRAIL-induced mutagenesis, we used CRISPR/Cas9 gene-editing to generate TK6 derivatives lacking CAD expression (Figure 1a). A ligation-mediated quantitative PCR method 'ApoqPCR'^40^ revealed that the level of apoptotic DNA was over fourfold higher in TRAIL-treated cells compared with untreated cells, however, hardly any fragmented DNA was detected in TRAIL-treated CAD KO lines (Figure 1b), hence CAD was the primary nuclease responsible for apoptotic DNA fragmentation in this context. Flow cytometric detection of cells bearing phosphorylated H2AX (*γ*H2AX) was used to quantitate the percentage of cells experiencing DNA damage. Exposure to TRAIL provoked a dose-dependent increase in the proportion of cells containing *γ*H2AX in control cells, but not in the CAD KO lines (Figure 1c). We also explored whether this CAD-dependent DNA damage correlated with mutations at the HPRT locus as measured by the emergence of 6TG-resistant (6TG^R^) cells. Sensitivity to TRAIL was similar in the cells containing and lacking CAD (Figures 1d and e). An increase in the HPRT mutation frequency was observed following TRAIL treatment of control cells, however, exposure to TRAIL did not provoke mutations in CAD KO cells (Figure 1f). Interestingly, cells lacking CAD also experienced less spontaneous mutagenesis at the HPRT locus than the parental cells.

**Figure 2:**
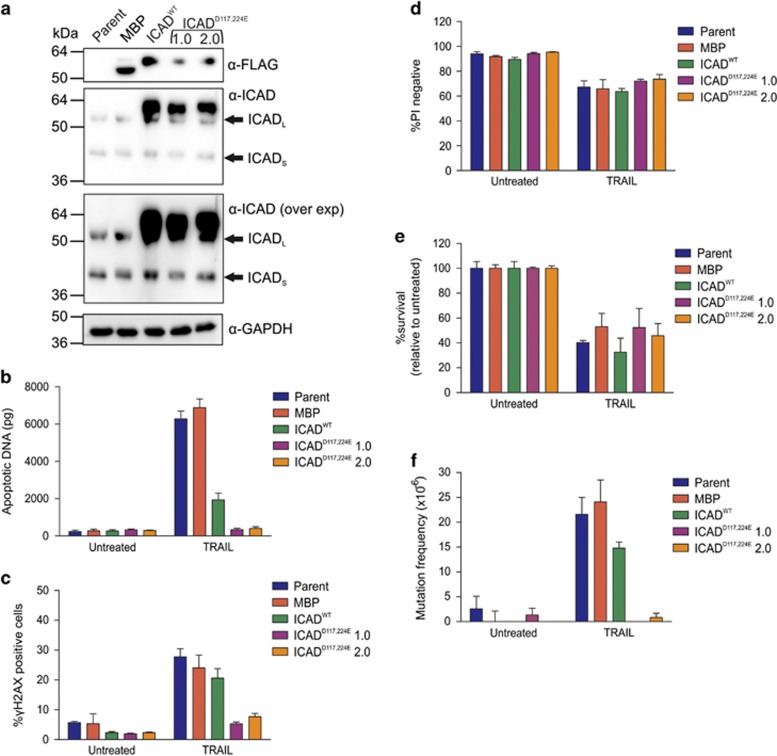
Overexpression of mutant ICAD prevents TRAIL mutagenesis. (**a**) ICAD, FLAG and GAPDH protein expression in parental cells and clones expressing FLAG-tagged constructs were assessed by immunoblot. (**b**) Cells were treated with no drug or 300 ng/ml TRAIL for 24 h then harvested. DNA was extracted for DNA fragmentation analysis by LM-qPCR detection. (**c**) Cells were treated with TRAIL for 5 h and levels of *γ*H2AX protein quantitated by flow cytometry. Cells were treated with 300 ng/ml TRAIL for 24 h then harvested. Propidium iodide was added to some cells to determine acute cell death (**d**), whereas clonogenicity assays were performed on other cells to determine the proportion of cells maintaining clonogenic competency after treatment (**e**). Surviving cells were grown in 6TG to select for the emergence of any HPRT mutants (**f**). Error bars represent mean±S.E.M. from three independent experiments

To further validate the mutagenic property of CAD, TK6 stable lines overexpressing an uncleavable mutant form of FLAG-tagged ICAD (ICAD^D117E, D224E^)^18^ were generated alongside clones overexpressing tagged wild-type ICAD (ICAD^WT^) and maltose-protein (MBP) (Figure 2a). TRAIL exposure failed to trigger significant DNA fragmentation or H2AX phosphorylation in cells expressing mutant ICAD, implying CAD remained inactive in these cells (Figures 2b and c). Overexpression of ICAD^WT^ reduced DNA fragmentation about threefold; consistent with a previous observation that increased expression of ICAD reduced CAD activity.^18^ Survival after TRAIL exposure reached similar levels in all lines tested (Figures 2d and e). TRAIL provoked HPRT mutations in parental, MBP and (to a lesser extent) ICAD^WT^ cells, but not in mutant ICAD cells (Figure 2f). These data demonstrate that CAD is essential for TRAIL-induced mutagenesis.

### TRAIL mutations occur because of executioner caspase activation

**Figure 3:**
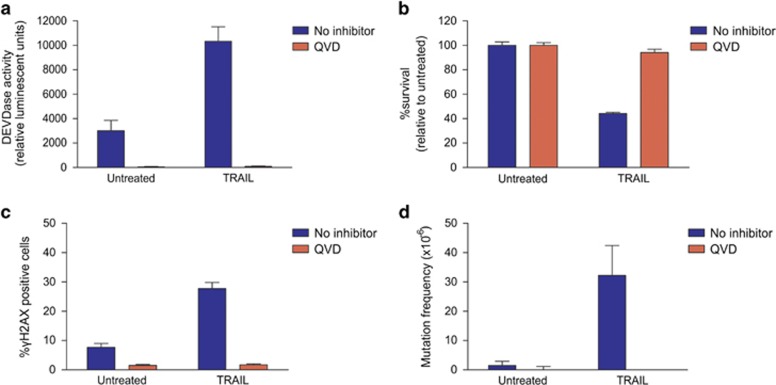
Caspase inhibition by QVD prevents TRAIL mutagenesis. (**a**) TK6 cells were incubated with no inhibitor or 10 *μ*M QVD then treated with 300 ng/ml TRAIL for 5 h. Caspase activity was assessed by luminescent detection using the Caspase-3/-7 Glo reagent. (**b**) Pre-treated cells were treated with 300 ng/ml TRAIL for 24 h. Clonogenicity assays were performed to determine the proportion of cells maintaining clonogenic competency after treatment. (**c**) Cells pre-treated with no inhibitor or 10 *μ*M QVD were treated with 300 ng/ml TRAIL for 5 h and *γ*H2AX-positive cells were detected by flow cytometry. (**d**) Surviving cells were grown in 6TG to select for the emergence of any HPRT mutants. Error bars represent mean±S.E.M. from three independent experiments

The data above indicate that CAD is essential for TRAIL mutagenesis, so we postulated that caspases are crucial for transmitting the mutagenic signal of TRAIL (by activating CAD). To test this, the pan caspase inhibitor Q-VD-OPh (QVD) was used to chemically inhibit caspase activity upon TRAIL treatment (Figure 3a). Caspase inhibition prevented cell death (Figure 3b), phosphorylation of H2AX (Figure 3c) and HPRT mutations (Figure 3d) associated with TRAIL treatment, confirming that activated caspases are required for TRAIL-mediated mutagenesis.

**Figure 4:**
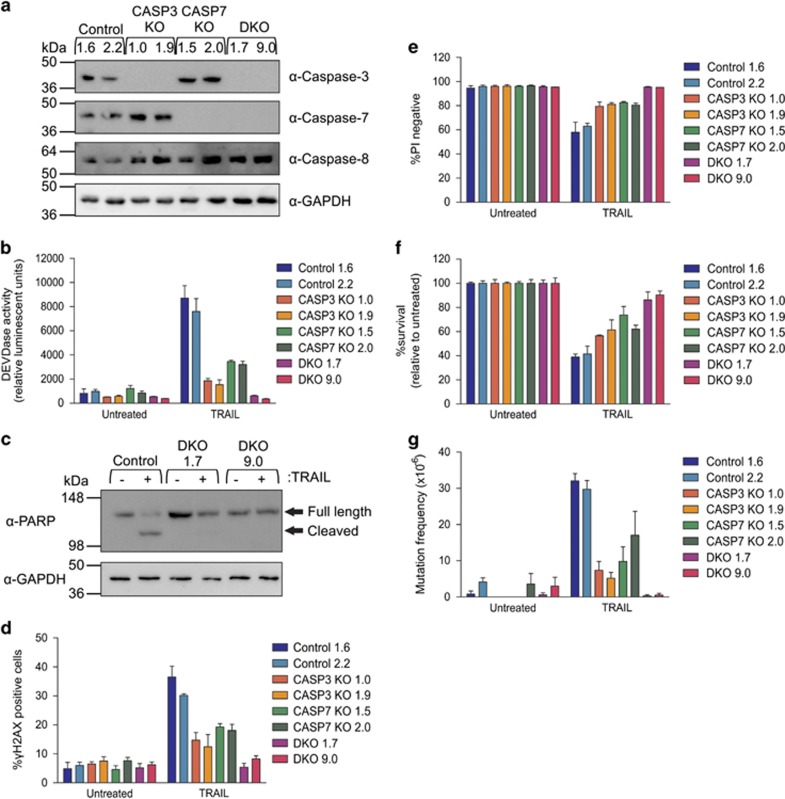
Executioner caspases are required for TRAIL mutagenesis. (**a**) Caspase-3, -7 and -8 protein expression in control, single knockout and double knockout cells was assessed by immunoblot. Probing for GAPDH was used to indicate loading. (**b**) Cells were treated with 300 ng/ml TRAIL for 5 h. Caspase activity was assessed by luminescent detection using Caspase-3/-7 Glo reagent. (**c**) Cells were treated with 300 ng/ml TRAIL for 4 h and lysates probed for PARP cleavage or GAPDH by immunoblot. (**d**) Cells were treated with 300 ng/ml TRAIL for 5 h and *γ*H2AX-positive cells were detected by flow cytometry. Cells were treated with no drug or 300 ng/ml TRAIL for 24 h. Propidium iodide was added to cells to assess for percentage of cells with damaged membranes by flow cytometry (**e**), whereas clonogenicity assays were performed on other cells to determine the proportion of cells maintaining clonogenic competency after treatment (**f**). Surviving cells were grown in 6TG to select for the emergence of any HPRT mutants (**g**). Error bars represent mean±S.E.M. from three independent experiments

We next wanted to ascertain whether executioner caspases, which were reported to most efficiently cleave ICAD to release CAD,^41^ are the essential caspases in this mutagenic process. Overexpression of a caspase-8 inhibitor, CrmA, prevented TRAIL-induced mutations^11^ (Supplementary Figure 1), demonstrating that TRAIL-mediated mutagenesis is caspase-8 dependent, however, those data did not preclude the involvement of downstream caspases. To model this, TK6 knockouts of either caspase-3 (CASP3 KO) or -7 (CASP7 KO) or both (DKO) were generated (Figure 4a). All lines retained caspase-8 expression. Exposure to TRAIL caused rapid DEVDase activity in control cells, but this was reduced in cells lacking either executioner caspase and was abolished when both caspases were absent (Figure 4b), and TRAIL failed to promote cleavage of PARP in the absence of executioner caspases (Figure 4c). TRAIL triggered H2AX phosphorylation in fewer CASP7 KO and CASP3 KO cells than wild-type cells, but a similar low proportion of treated and untreated DKO cells bore *γ*H2AX (Figure 4d), mirroring the levels of DEVDase activity. Although the membranes of DKO cells remained intact after 24- h exposure to TRAIL (Figure 4e), clonogenic survival was reduced by about 15% in these cells (Figure 4f) possibly because of impaired mitochondrial function. Slightly fewer TRAIL-treated single KO cells lost clonogenic potential than controls. The frequency of mutations in the absence of executioner caspases also reflected the level of DEVDase activity: removal of both caspase-3 and -7 prevented TRAIL mutations. However, the presence of at least one of these caspases enabled TRAIL treatment to yield a small number of 6TG^R^ clones (Figure 4g). Mutation frequencies (and the level of DNA damage) seemed slightly higher in CASP7 KO cells than CASP3 KO cells.

### TRAIL mutations can occur with or without mitochondrial amplification

**Figure 5:**
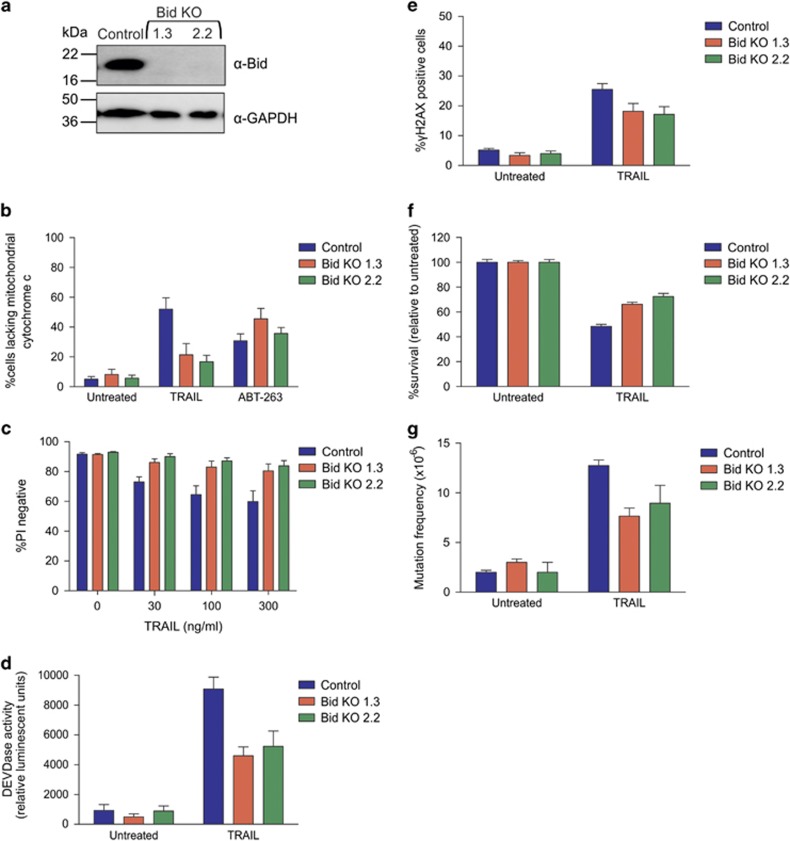
TRAIL induces slightly fewer mutations in the absence of mitochondrial apoptotic signaling. (**a**) Bid protein expression in parent (control) and Bid knockout cells was assessed by immunoblot. The blot was reprobed with an antibody to GAPDH to indicate loading. (**b**) Cells were treated with no drug, 300 ng/ml TRAIL or 5 *μ*M ABT-263 for 4 h. After permeabilizing the plasma membrane, and washing to remove cytosolic contents, cells were stained with cytochrome *c* primary antibody then FITC-labeled secondary antibody to determine the percentage of cells lacking residual (mitochondrial) cytochrome *c*. (**c**) Cells were treated with TRAIL for 24 h and propidium iodide added to cells to assess the percentage of cells with damaged membranes. Cells were treated with 300 ng/ml TRAIL for 5 h then (**d**) caspase activity assessed by luminescent detection of Caspase-3/-7 Glo reagent or (**e**) *γ*H2AX-positive cells were detected by flow cytometry. (**f**) Clonogenicity assays were performed on cells exposed to 300 ng/ml TRAIL for 24 h to determine the proportion of cells maintaining clonogenic competency after treatment, whereas (**g**) surviving cells were grown in 6TG to select for the emergence of any HPRT mutants. Error bars represent mean±S.E.M. from three independent experiments

TRAIL promotes caspase-8 activation and can activate executioner caspases through direct cleavage by caspase-8 (type I) or via Bid-mediated MOMP leading to caspase-9 cleavage (type II).^13, 42^ TK6 cells lacking expression of Bid were generated (Figure 5a) to assess if TRAIL mutations occurred through direct caspase-8-mediated activation of caspases-3 and -7 (and hence CAD) or if caspase-9 contributes to some or all of the mutations by proteolytically activating the executioner caspases. Mitochondrial factors such as cytochrome *c* are released upon Bax/Bak activation, promoting apoptosome formation to activate caspase-9.^43^ We determined the proportion of cells retaining mitochondrial cytochrome *c* after treatment, to assess the extent of mitochondrial amplification of extrinsic apoptotic signaling following TRAIL treatment. Treatment with the BH3 mimetic ABT-263 promoted intrinsic apoptotic signaling, as detected by an increase in cells lacking mitochondrial cytochrome *c* (Figure 5b). This was also observed after treatment with TRAIL, implying TRAIL signaling in TK6 cells compromises the mitochondrial outer membrane integrity and presumably activates caspase-9. TRAIL killed around 20% fewer Bid KO cells than control cells (Figure 5c). The proportion of Bid KO cells treated with TRAIL exhibiting MOMP was minimal (Figure 5b) suggesting most contained intact mitochondria and implying direct signaling by caspase-8 in the absence of Bid. The level of caspase-3/-7 activation, and the proportion of *γ*H2AX-positive cells following incubation with TRAIL, were reduced by about one-third in Bid KO cells compared with control cells (Figures 5d and e). Clonogenic survival was around 10–15% higher in cells lacking Bid compared with controls after TRAIL exposure (Figure 5f), suggesting that mitochondrial signaling further promotes clonogenic death. TRAIL exposure promoted mutations in Bid KO cells, although the frequency was reduced by about one-third (Figure 5g). Approximately one-third of TRAIL-induced mutations may be attributed to executioner caspase activation caused by caspase-9, whereas the remaining two-thirds are most likely directly because of caspase-3/-7 activation by caspase-8.

### Caspases and CAD are important for mutations caused by vincristine

**Figure 6:**
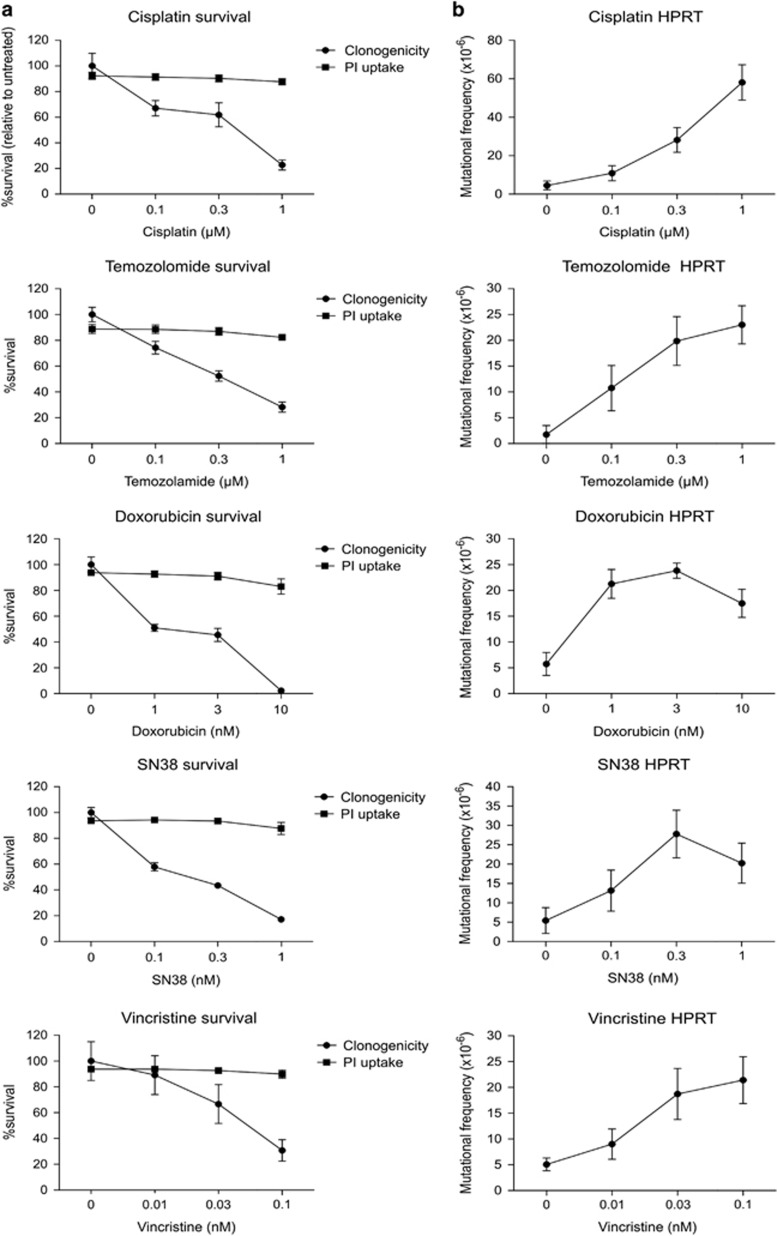
Quantitation of HPRT mutation frequencies following treatment with chemotherapy drugs. Cells were treated with specified concentration of drug for 24 h. (**a**) Propidium iodide and clonogenicity assays were performed to determine the proportion of surviving cells. (**b**) Surviving cells were grown in 6TG to select for the emergence of any HPRT mutants. Error bars represent mean±S.E.M. from three independent experiments

Classical ‘DNA damaging’ chemotherapy drugs can provoke mutations in surviving cells, potentially promoting therapy-related second cancers.^44^ Exposure to chemotherapy drugs can stimulate intrinsic apoptotic pathways that, like TRAIL signaling, involve the activation of caspases and CAD.^45^ We therefore tested the hypothesis that a proportion of chemotherapy-induced mutations might also be because of the mis-repair of CAD-mediated double-strand DNA breaks. We first quantitated the mutagenic potential of representative drugs from clinically used chemotherapy classes. Cells experienced a dose-dependent decrease in clonogenic survival after 24-h exposure to all drugs, although membranes of most cells remained intact (Figure 6a). Surviving cells were then incubated in 6TG to assess the emergence of cells with HPRT mutations. All drugs increased the frequency of 6TG^R^ cells (Figure 6b), with cisplatin being the most mutagenic.

**Figure 7:**
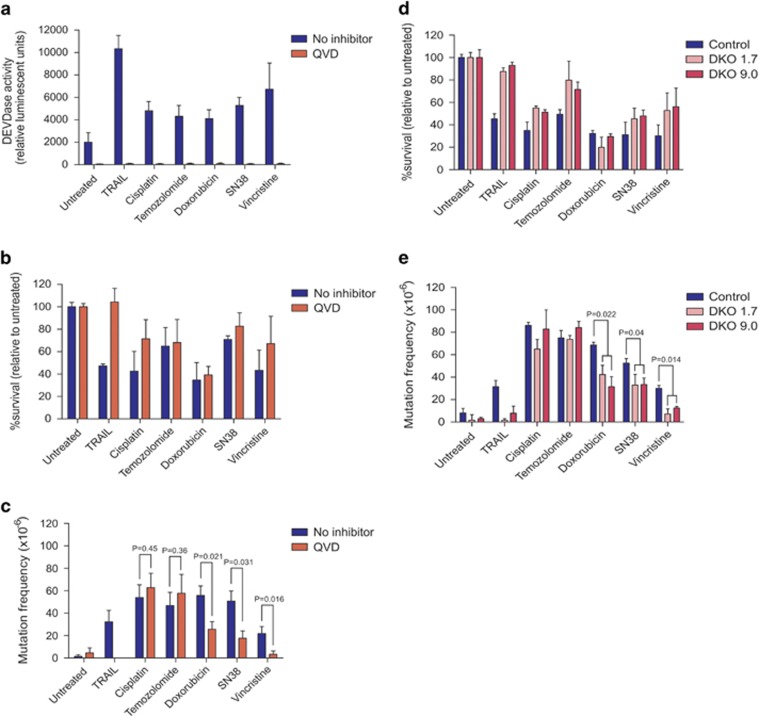
Determination of mutation frequencies in cells treated with chemotherapy drugs following caspase inhibition. TK6 cells were incubated with no inhibitor or 10 *μ*M QVD then treated with the following drug doses: 300 ng/ml TRAIL, 0.7 *μ*M cisplatin, 0.5 *μ*M temozolomide, 3 nM doxorubicin, 0.5 nM SN38 or 0.07 nM vincristine. (**a**) Caspase activity was assessed by luminescent detection of Caspase-3/-7 Glo reagent after 5 h. After 24- h treatment, (**b**) clonogenicity assays were performed to determine the proportion of cells maintaining clonogenic competency after treatment, whereas (**c**) surviving cells were grown in 6TG to select for the emergence of any HPRT mutants. (**d**) Clonogenicity and (**e**) HPRT mutation assays were repeated in CASP3/7 DKO lines in the same manner. Error bars represent mean±S.E.M. from at least three independent experiments. Two-sided *T*-tests were used to calculate *P*-values

Cells were exposed to chemotherapy drugs or TRAIL following incubation with (or without) QVD. All drugs caused an increase in DEVDase activity, which was completely inhibited by QVD (Figure 7a). QVD pre-treatment prevented TRAIL-induced clonogenic death but did not promote a marked increase in clonogenic potential after exposure to the other drugs (Figure 7b). This probably reflects mitochondrial damage associated with intrinsic apoptotic signaling triggered by these chemotherapy drugs, which was demonstrated to be sufficient to prevent clonogenic survival even in cells that lack active caspases.^46^ Treatment with concentrations of drugs that abolished clonogenic survival of about 40–60% of the cells increased the HPRT mutation frequency in surviving cells (Figure 7c). Strikingly, QVD pre-treatment prevented vincristine-induced mutagenesis. Mutation frequencies in QVD pre-treated cells treated with doxorubicin or SN38 were also significantly reduced. QVD did not significantly change frequencies of mutagenesis following cisplatin or temozolomide exposure. This trend was also observed using CASP3/7 DKO cells (Figures 7d and e). These assays demonstrate that caspases are required for vincristine (and TRAIL) mutagenesis, and for a proportion of the mutations provoked by doxorubicin and SN38. CrmA expression did not affect clonogenic potential or mutation frequencies following exposure to vincristine, doxorubicin or SN38 (Supplementary Figure 1), consistent with caspase-9 (rather than -8) having the initiator caspase role in these contexts.

**Figure 8:**
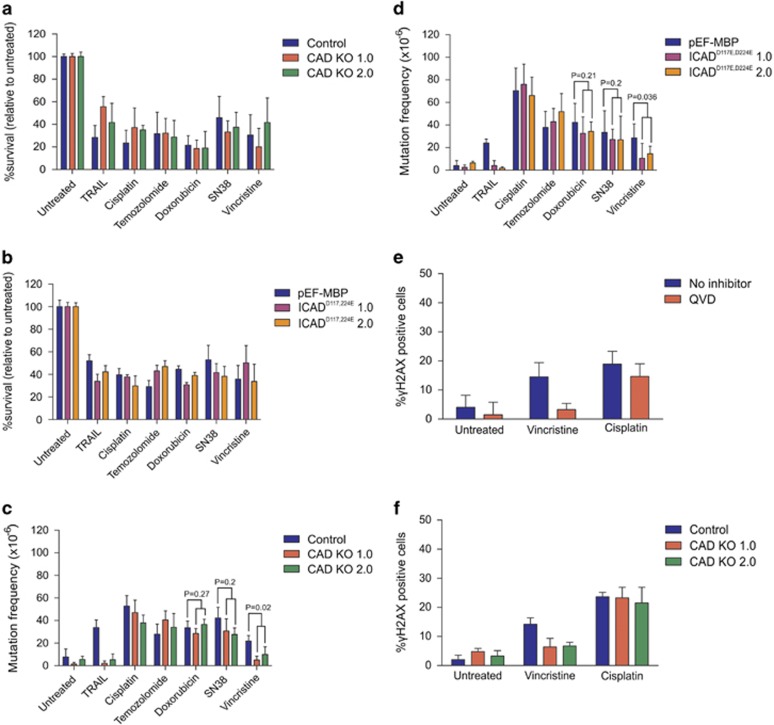
Determination of mutation frequencies in cells lacking CAD activity treated with chemotherapy drugs. (**a**-**d**) Control, CAD KO and ICAD-expressing cells were incubated with 300 ng/ml TRAIL, 0.7 *μ*M cisplatin, 0.5 *μ*M temozolomide, 3 nM doxorubicin, 0.5 nM SN38 or 0.07 nM vincristine for 24 h. Clonogenicity assays were performed to determine the proportion of cells maintaining clonogenic competency after treatment, (**a** and **b**) whereas surviving cells were grown in 6TG to select for the emergence of any HPRT mutants (**c** and **d**). (**e**) TK6 cells incubated with no inhibitor or 10 *μ*M QVD or (**f**) CAD KO cells were treated with 0.07 nM vincristine or 0.7 *μ*M cisplatin. After 5- h drug exposure, the level of *γ*H2AX protein was quantitated by flow cytometry. Error bars represent mean±S.E.M. from at least three independent experiments. Two-sided *T*-tests were used to calculate *P*-values

To determine whether the observed reduction in mutations in cells lacking executioner caspase activity was because of suppression of caspase-mediated CAD activation, CAD KO and ICAD mutant lines were exposed to the same drug panel. All lines experienced similar levels of clonogenic death upon treatment with the selected doses of each drug (Figures 8a and b). Interestingly, lack of CAD activity only achieved a marked reduction in mutation frequencies following vincristine and TRAIL treatment (Figures 8c and d). Vincristine caused an increase in *γ*H2AX-positive cells, but the proportion of vincristine-treated cells bearing *γ*H2AX was lower when caspases were inhibited (Figure 8e) or CAD was absent (Figure 8f). In contrast, cisplatin provoked H2AX phosphorylation in similar proportions of cells containing or lacking active caspases or CAD (Figures 8e and f).

## Discussion

Recognition of DNA damage stimulates an apoptotic response in cells exposed to conventional chemotherapy drugs, however, in contexts where cell death is not achieved, cells may mis-repair this damage, potentially facilitating oncogenic mutagenesis. The mechanisms underlying this have been postulated to include the formation of lesions that are commonly mis-repaired or regional 'hotspots' for DNA damage.^8, 47^ Ligation of death receptors by agonists such as TRAIL can activate DNA repair pathways as well as provoke mutations in surviving cells,^11, 34^ despite not needing to damage DNA in order to stimulate an apoptotic response. Mis-repair of double-stranded DNA breaks upon apoptotic signaling could be because of endonucleases, such as CAD^11^ or EndoG.^37^ Trapped topoisomerase-I cleavage complexes^48^ have also been described in cells treated with TRAIL and other apoptotic stimuli. The first goal of this study was therefore to conclusively ascertain whether or not TRAIL provokes mutations via CAD-dependent mechanisms.

Two independent techniques were used to eliminate CAD activity in TRAIL-treated cells. Knockout of CAD and overexpression of uncleavable mutant ICAD (to limit the release of active CAD) suppressed TRAIL-induced DNA damage and the ensuing mutations. Fewer spontaneous HPRT mutations appeared in cells lacking active CAD, which may reflect some basal level of CAD-mediated DNA damage.^49^ We failed to detect mutations in TRAIL-treated cells when caspases were inhibited by QVD, indicating the requirement for caspases. We also sought to identify the caspases that were essential in delivering the mutagenic signal of TRAIL. Inhibition of caspase-8, or knockout of both caspase-3 and -7 prevented any TRAIL mutations, whereas mutation frequencies were reduced when only one executioner caspase was present. Slightly fewer mutations were observed in CASP3 KO lines compared with CASP7 KO lines, which could reflect a higher ICAD cleavage rate by caspase-3 than caspase-7.^20, 41^ Caspase-9 was not essential for TRAIL-mediated mutagenesis as Bid KO cells, which lacked the cytosolic cytochrome *c* required for apoptosome formation, still acquired mutations, albeit with reduced frequency.

We therefore conclude that sublethal TRAIL signaling promotes caspase-8-dependent activation of executioner caspases-3 and -7, to activate CAD and generate double-strand DNA breaks. Mis-repair of this damage (most likely because of NHEJ) can facilitate the formation of mutations, such as deletions,^36^ in surviving cells. Our study provides further evidence that cells are able to survive and proliferate despite containing active caspases and CAD, consistent with previous illustrations of non-apoptotic roles of caspases and CAD.^50, 51, 52^

Our observation that TRAIL-mediated apoptotic signaling could cause mutations in surviving cells via CAD-mediated double-strand DNA breaks led us to speculate that chemotherapy-induced apoptosis may also promote mutations via this pathway. Cisplatin and temozolomide were mutagenic regardless of caspase or CAD activity, arguing that mutations provoked by these drugs result from their direct effects on DNA. In contrast, mutations provoked by vincristine were CAD and caspase-3/-7 dependent. Microtubule-targeting drugs like vincristine promote rapid caspase activation via intrinsic apoptotic pathways as a result of mitotic catastrophe.^53^ Our data imply that the defects in chromosomal segregation triggered by microtubule destabilizing poisons like vincristine are not directly mutagenic, rather we ascribe the genotoxicity associated with this process to the sublethal apoptotic signaling that it provokes in cells – specifically caspase and CAD activation. Previous research has implicated an essential role for caspases and CAD in DNA damage induced by other spindle poisons in cells experiencing prolonged mitotic arrest.^29, 30^ Those assays, however, did not distinguish DNA damage in dying or surviving cells. This study revealed that vincristine provoked caspase-/CAD-dependent mutagenesis in surviving, clonogenically competent cells. Apoptotic signaling triggered by mitotic catastrophe has been proposed to maintain genomic stability by preventing the survival of aneuploid cells,^54^ however, our data reveal that the ability of anti-mitotic agents to provoke sublethal caspase and CAD activity can create mutations, thus instigation of mitotic catastrophe may not necessarily be onco-suppressive.

Some of the mutations that emerged following topoisomerase inhibition by doxorubicin and SN38 were because of caspases, although CAD status did not significantly affect the mutagenicity of these stimuli. Doxorubicin can also induce mitotic arrest.^55^ If caspase-dependent mutagenesis during prolonged mitotic arrest (and the ensuing mitotic catastrophe) caused by spindle poisons holds true for other cytotoxic drugs that also promote mitotic arrest, like doxorubicin, this may account for our observation that reducing caspase activity protected some cells from doxorubicin-mediated mutagenesis. Further work will be needed to explore a potential caspase-dependent but CAD-independent mutagenesis pathway that our data imply can be triggered by doxorubicin (and SN38), but it is possible that other apoptotic endonucleases are responsible for a subset of the DNA damage caused by these drugs. EndoG is mitochondrially localized in healthy cells but translocates to the nucleus to facilitate DNA fragmentation upon MOMP.^56^ EndoG was implicated in caspase-dependent DNA damage because of some sublethal apoptotic stimuli,^37, 56^ however, MLLbcr recombination caused by aphidicolin appeared to be EndoG dependent but caspase independent.^57^ Doxorubicin was reported to promote the release of reactive oxygen species (ROS) in order to carry out apoptosis.^58, 59^ Caspase-mediated loss of mitochondrial integrity has been linked to the generation of ROS, which can be potentially oncogenic.^59, 60^ Therefore, stimuli that promote apoptotic mitochondrial damage (such as inhibition of topoisomerase proteins) may stimulate EndoG- or ROS-mediated mutagenesis independent of CAD. Trapping of topoisomerase-I complexes within oxidative DNA sites can occur in response to genotoxic DNA nicks^61^ and also in apoptotic cells.^48, 62^ These complexes can also be stabilized in the presence of topoisomerase-I inhibitors, such as camptothecin or SN38, to promote double-strand DNA breaks upon incorrect replication or transcription.^63^ Hence, caspases may augment the number of stabilized trapped topoisomerase-I complexes caused by SN38, which may explain the contribution of caspases-3 and -7 to SN38-induced mutations.

This study documents the critical importance of caspases and CAD to mutagenesis by TRAIL and vincristine, and attributes a proportion of the mutagenic activity of doxorubicin and SN38 to caspase-dependent mechanisms. Genome sequencing of DNA breaks caused by apoptotic nucleases, like CAD, revealed that they localized at actively transcribed genes, particularly at genes frequently translocated in human cancer.^26^ The involvement of sublethal apoptotic signaling in mutagenesis induced by death receptor agonists, mitotic poisons and topoisomerase inhibitors may help define the oncogenic potential of these drugs.

## Materials and methods

### Reagents and cell lines

TK6^64^ and Jurkat cells were grown in RPMI-1640 containing HEPES buffer (Invitrogen, Carlsbad, CA, USA) supplemented with 10% heat inactivated FBS (Invitrogen). HEK-293T cells were cultured in Dulbecco’s modified Eagle's medium with high glucose (Invitrogen) supplemented with 10% heat inactivated FBS. All cells were grown at 37 °C in air supplemented with 5% CO_2_.

Drugs in this study were recombinant human sTRAIL/Apo2L (Peprotech, Rocky Hill, NJ, USA), doxorubicin (Sigma, St.Louis, MO, USA), ABT-263 (Selleck Chemicals, Houston, TX, USA), cisplatin (Sigma), temozolomide (Selleck Chemicals), SN38 (Selleck Chemicals), vincristine (Selleck Chemicals), staurosporin (Sigma), doxycycline (Sigma) and 6-thioguanine (6TG) (Sigma). The following antibodies were used: rabbit anti-CAD (FL-338) (Santa Cruz Biotechnology, Dallas, TX, USA; sc-8342), mouse anti-ICAD (MBL International, Woburn, MA, USA; M037-3), mouse anti-FLAG (M2) (Sigma; #3165), goat anti-Bid (R&D Systems, Minneapolis, MN, USA; AF860), mouse anti-caspase-3 (clone 19) (BD Biosciences, San Jose, CA, USA; #610323), rabbit anti-caspase-7 (Cell Signaling Technology, Danvers, MA, USA; #9294S), mouse anti-caspase-8 (Cell Signaling Technology; #9746), mouse anti-PARP (46D11) (Cell Signaling Technology; #9532), rabbit anti-H2AX (Ser 139) clone 20E3 (Cell Signaling Technology; #9718), mouse anti-cytochrome c (BD Biosciences; #556342), mouse anti-GAPDH (Merck Millipore, Mellerica, MA, USA; #MAB374), goat anti-rabbit-FITC (Merck Millipore), donkey anti-rabbit-HRP (GE Healthcare Life Sciences, Princeton, NJ, USA), rabbit anti-mouse-HRP (Sigma) and swine anti-goat-HRP (Southern Biotech, Birmingham, AL, USA).

### Plasmids

Plasmids bearing human wild-type ICAD or uncleavable mutant ICAD^D117, D224E^ were custom made by GenScript (Piscataway, NJ, USA). This mutant was shown to be resistant to caspase cleavage.^18^ These open reading frames were cloned into the pEF vector^65^ along with a FLAG epitope to encode FLAG-tagged wild type ICAD (pEF-FLAG-ICAD^WT^) or mutant ICAD (pEF-FLAG-ICAD^D117E, D224E^). The pEF-FLAG-CrmA plasmid was previously described.^65^ A sequence encoding MBP was amplified from pMal-c2x (New England Biolabs, Ipswich, MA, USA) with the following primers: 5′-GTCGGATCCACCATGAAAATCGAAGAAGGTAAAC-3′ and 5′-TTAAGTTGGGTAACGCCAG-3′. The product was cut with *Bam*HI then ligated into FLAG-pEF^66^ that had been cut with *Bam*HI and dephosphorylated.

The pFUCas9mCherry and pFgh1tUTG plasmids^67^ were kindly provided by Hamsa Puthalakath and Marco Herold. For single guide RNA (sgRNA) plasmids, the following oligonucleotide pairs were annealed and ligated into pFgh1tUTG that had been cut with BsmB1 using published methods.^68^

*CAD* exon 2: 5′-TCCCTGTTCCCGACAACGCCGAGC-3′, 5′-AAACGCTCGGCGTTGTCGGGAACA-3′

*BID* exon 3: 5′-TCCCCGCAGAGAGCTGGACGCACT-3′, 5′-AAACAGTGCGTCCAGCTCTCTGCG-3′

*CASP3* exon 2: 5′-TCCCGGAAGCGAATCAATGGACTC-3′, 5′-AAACGAGTCCATTGATTCGCTTCC-3′

*CASP7* exon 2: 5′-TCCCGACCGGTCCTCGTTTGTACC-3′, 5′-AAACGACCGGTCCTCGTTTGTACC-3′.

### Generation of CRISPR/Cas9 knockout lines

CRISPR/Cas9 technology was used to generate knockout derivatives of the TK6 line via a doxycycline-inducible sgRNA vector system according to a described method.^68^ TK6 cells constitutively expressing the Cas9 endonuclease (TK6-Cas9) were generated by lentiviral transduction with the pFUCas9mCherry plasmid. These cells were then infected via lentiviral delivery with individual sgRNA plasmids to generate TK6 cells constitutively expressing Cas9 (mCherry positive) alongside a doxycycline-inducible sgRNA (GFP positive). For generating CASP-3/-7 double knockout (DKO) cells, TK6-Cas9 cells were simultaneously infected with lentiviral particles containing sgRNAs for both *CASP3* and *CASP7*.

Lentiviral particles containing plasmid were generated by infecting HEK-293 T cells with a cocktail of 2 *μ*g pCMV-R8.91 and 0.8 *μ*g pVSV-G packaging constructs, 1.2 *μ*g pFgh1tUTG plasmid containing the guide sequence and 12 *μ*l FuGene reagent (Promega, Fitchburg, WI, USA). Cells were cultured for 48 h then supernatant collected and filter sterilized. Five-hundred thousand TK6-Cas9 cells were seeded per well in six-well plates in the presence of 3 ml lentiviral supernatant containing polybrene, and spin-infected at 37 °C for 45 min at 2500 r.p.m. Cells were incubated overnight then resuspended in complete media and cultured for 3 days. A FACS Aria II (BD Biosciences) was used to single-cell sort for mCherry/GFP double-positive cells into 96-well plates containing 100 *μ*l media supplemented with 20% FBS and containing 1 *μ*g/ml doxycycline.

### Generation of mutant ICAD stable lines

Stable transfection of TK6 cells was carried out by Nucleofection using the SF Nucleofector kit (Lonza, Allendale, NJ, USA). Briefly, 10^6^ cells were transfected with 0.8 *μ*g plasmid using the Nucleofector SF solution according to the manufacturer’s instruction and the DN-100 program with a Nucleofector device (Lonza). Cells were then grown in RPMI/20% FCS media for 2 days then seeded at a density of 1000 cells per well in a 96-well plate in media containing 0.1 *μ*g/ml puromycin. Plates were incubated for 10–14 days then clones were expanded and characterized.

### Immunoblotting

Immunoblotting was conducted as previously described.^10^ Blotted membranes were incubated with block buffer (1% blocking reagent; Roche, Sydney, NSW, Australia) in PBS then probed with anti-CAD (1 : 500), anti-ICAD (1 : 500), anti-FLAG (1 : 1000), anti-Bid (1 : 200), anti-caspase-3 (1 : 1000), anti-caspase-7 (1 : 500), anti-caspase-8 (1 : 500), anti-PARP (1 : 500) or anti-GAPDH (1 : 5000) in block buffer. Horseradish peroxidase (HRP)-conjugated secondary antibodies (1 : 20 000 in block buffer containing 1% Triton X-100) were detected using SuperSignalWest Dura Extended Duration Substrate (Thermo Fisher Scientific, Vic, Australia).

### Acute and clonogenic survival assays

Treated cells (5x10^5^ cells per ml) were subjected to propidium iodide (PI) uptake and clonogenicity assays to measure survival. For PI uptake assays, cells were harvested and resuspended in 1 *μ*g/ml PI (Sigma) in PBS. Flow cytometry was used to quantitate the proportion of PI-positive cells using FACS Canto II (BD Biosciences). For clonogenicity assays, cells were harvested after treatment, washed once in PBS and counted. Cells were then seeded at an appropriate density in media containing 15% FCS and dispensed at 100 *μ*l per well into a round bottom 96-well plate. After 10–14 days, plates were scored for number of wells with growth, and cloning efficiency (CE) calculated using the formula: CE=-ln (proportion of wells lacking growth)/number of cells seeded per well.

### Caspase activity assay

DEVDase activity was measured after treatment using the Caspase-3/-7 Glo assay kit (Promega). Ten thousand cells were seeded in 96-well white plates in media alone or media containing drug to a final volume of 50 *μ*l and incubated for 6 h. In some experiments, cells were pre-treated for 1 h with 10 *μ*M of Q-VD-OPh (R&D Systems) before addition of drug. After treatment, 50 *μ*l of Caspase-3/-7 Glo solution was mixed into each well and plates incubated for 30 min at room temperature. Luminescence was recorded using a Spectromax M5 (Molecular Devices, Sunnyvale, CA, USA).

### *γ*-H2AX detection by flow cytometry

Detection and quantitation of cells bearing *γ*-H2AX protein was assayed as conducted previously,^10^ except that a 1 : 250 dilution of each antibody was used.

### HPRT assays

HPRT assays were conducted according to a previously published method.^10^ Mutation frequency was calculated using the formula: mutation frequency=cloning efficiency (6TG selection)/plating efficiency (non-selective condition).

### Cytochrome c detection by flow cytometry

This assay was performed according to a previously published method^42^ except that membranes were permeabilized in 100 *μ*g/ml digitonin (Sigma) diluted in 80 nM KCl/PBS then fixed in 4% paraformaldehyde (Sigma) for 20 min at room temperature.

### Detection of apoptotic DNA by LM-qPCR

Ligation-mediated qPCR was used to quantitate amount of apoptotic DNA present in cells according to a published 'ApoqPCR' protocol^40^ with some changes. Apoptotic standard DNA (six serial fourfold dilutions beginning from 9.26 ng/ml) was generated from Jurkat cells treated with 10 *μ*M staurosporin for 6 h. For test samples, 5x10^5^ TK6 cells were treated with or without drug for 24 h. Genomic DNA was extracted using the DNeasy Blood and Tissue kit (Qiagen, Germantown, MD, USA). Conditions for annealing/ligation reactions were the same as previously described. For LM-qPCR, reactions contained 7.5 *μ*l diluted annealed/ligated products, 12.5 *μ*l OneTaq Hot Start 2X Master Mix with standard buffer (New England Biolabs, Ipswich, MA, USA), 2 *μ*M 24-mer oligonucleotide, and 0.4 X SYBR Green I (Invitrogen) to a final reaction volume of 25 *μ*l. Fragments were amplified using a Stratagene Mx3000P qPCR machine (Agilent Technologies, Santa Clara, CA, USA) according to the same conditions. An apoptotic standard curve was generated from known apoptotic standards and quantitation of unknown apoptotic test DNA interpolated using GraphPad Prism software (GraphPad Software Inc., La Jolla, CA, USA).

### Statistics

*P*-values were calculated using two-sided, unpaired *T*-tests to compare mutation frequencies. Where two clones represent a particular phenotype, frequencies of both clones were combined and compared with control cells per the condition.
