# Supplementary material for: Executioner caspases and CAD are essential for mutagenesis induced by TRAIL or vincristine
Source: Cell Death Dis. 2017 Oct 5;8(10):e3062–. doi: 10.1038/cddis.2017.454 (PMC5680576; doi:10.1038/cddis.2017.454)
Supplement: Supplementary Figure 1 [file cddis2017454x1.pdf]

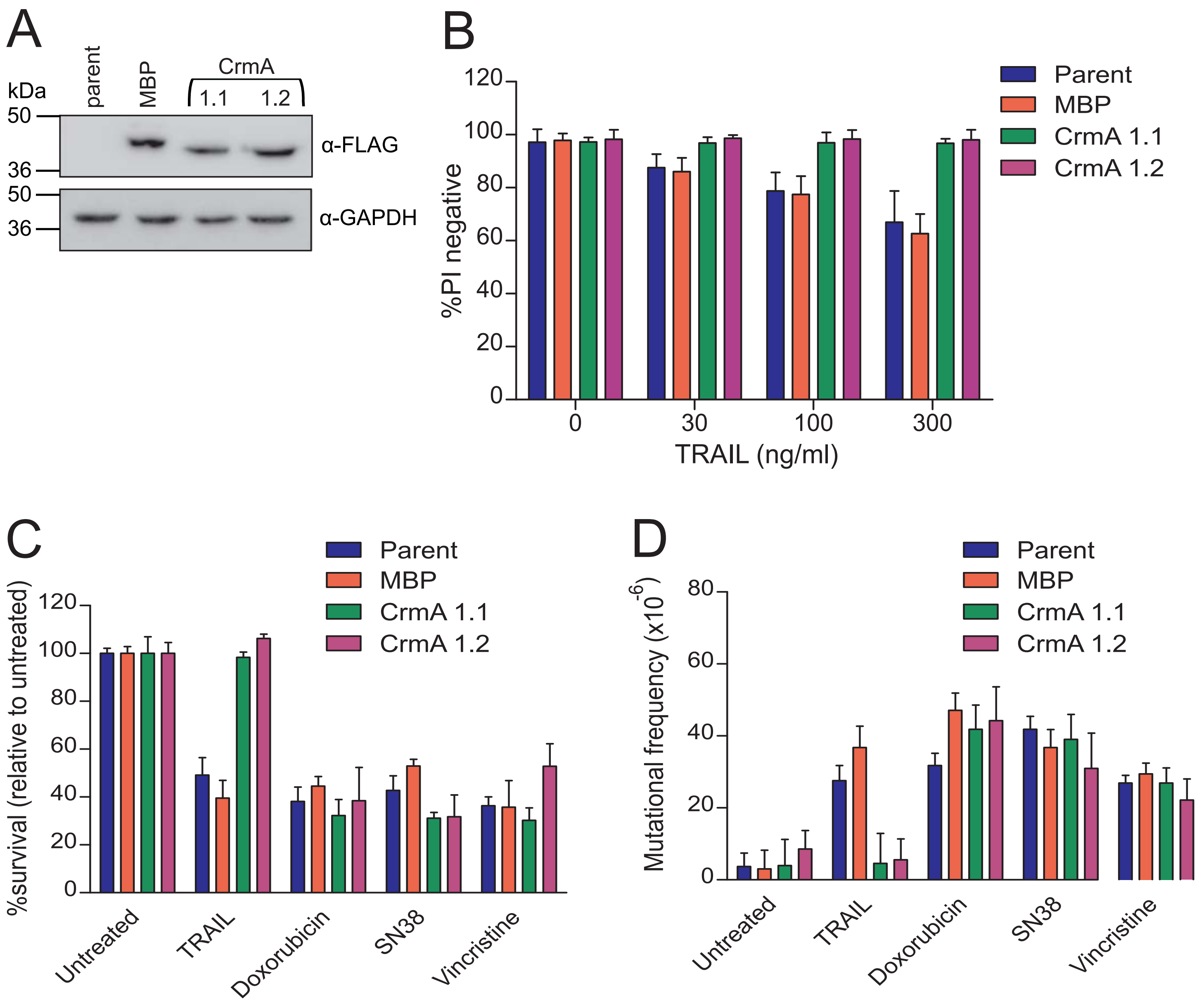

Supplementary Figure 1 CrmA expression prevents apoptosis and mutagenesis following exposure to TRAIL but not chemotherapy drugs. (a) Parental TK6 cells and stable TK6 transfectants expressing FLAG-tagged Maltose Binding Protein (MBP) or FLAG-CrmA were lysed, then transgene and GAPDH expression were assessed by immunoblotting. (b) Cells were exposed to the specified concentrations of TRAIL for 24 h then acute death was quantitated by propidium iodide uptake. (c-d) Cells were treated for 24 h with 300 ng/ml TRAIL, 3 nM doxorubicin, 0.5 nM SN38 or 0.07 nM vincristine then clonogenicity assays were performed to determine the proportion of cells maintaining clonogenic competency after treatment (c) and surviving cells were grown in 6TG to select for the emergence of HPRT mutants (d). Error bars represent mean ± SEM from three independent experiments.
